# Composition of gut microbiota in patients with toxigenic *Clostridioides (Clostridium) difficile*: Comparison between subgroups according to clinical criteria and toxin gene load

**DOI:** 10.1371/journal.pone.0212626

**Published:** 2019-02-20

**Authors:** Sung-Hee Han, Joowon Yi, Ji-Hoon Kim, SangWon Lee, Hee-Won Moon

**Affiliations:** 1 BioCore Co. Ltd., Biotechnology, Yongin, Republic of Korea; 2 Department of Laboratory Medicine, Konkuk University School of Medicine, Seoul, Republic of Korea; 3 BioCore Co. Ltd., R&D Center, Seoul, Republic of Korea; 4 College of Veterinary Medicine, Konkuk University, Seoul, Republic of Korea; Institut Pasteur, FRANCE

## Abstract

Data concerning the human microbiota composition during *Clostridioides (Clostridium) difficile infection* (CDI) using next-generation sequencing are still limited. We aimed to confirm key features indicating *tcdB* positive patients and compare the microbiota composition between subgroups based on toxin gene load (*tcdB* gene) and presence of significant diarrhea. Ninety-nine fecal samples from 79 *tcdB* positive patients and 20 controls were analyzed using 16S rRNA gene sequencing. Chao1 index for alpha diversity were calculated and principal coordinate analysis was performed for beta diversity using Quantitative Insights into Microbial Ecology (QIIME) pipeline. The mean relative abundance in each group was compared at phylum, family, and genus levels. There were significant alterations in alpha and beta diversity in *tcdB* positive patients (both colonizer and CDI) compared with those in the control. The mean Chao1 index of *tcdB* positive patients was significantly lower than the control group (*P*<0.001), whereas there was no significant difference between *tcdB* groups and between colonizer and CDI. There were significant differences in microbiota compositions between *tcdB* positive patients and the control at phylum, family, and genus levels. Several genera such as *Phascolarctobacterium*, *Lachnospira*, *Butyricimonas*, *Catenibacterium*, *Paraprevotella*, *Odoribacter*, and *Anaerostipes* were not detected in most CDI cases. We identified several changes in the microbiota of CDI that could be further evaluated as predictive markers. Microbiota differences between clinical subgroups of CDI need to be further studied in larger controlled studies.

## Introduction

*Clostridioides (Clostridium) difficile* is a common cause of antibiotic-associated colitis. CDI rates have plateaued in the United States since about 2010 although rates have declined remarkably in England and other parts of Europe since their peak before 2010 [1–4]. CDI has a broad spectrum of clinical features, ranging from mild diarrhea to severe diseases such as toxic megacolon. Although toxigenic *C*. *difficile* is detected in patient samples, many patients do not meet the criteria for significant diarrhea [5–7].

The most important risk factor for CDI is antibiotic use [8]. In susceptible hosts, microbiota-mediated colonization resistance is diminished partly by a reduction in the diversity of the gut microbiota caused by antibiotics [1, 8–11]. After antibiotic treatment for CDI, there is a phase of the restoration of normal microbiota, which itself averts recurrence of CDI [8, 12]. Thus, recently developed therapy, such as fecal microbiota transplantation (FMT), tries to restores gut microbiota diversity instead of the direct eradication of the pathogen [13, 14].

Decreased diversity and alteration of the gut microbiota composition in CDI has been shown in previous studies using various techniques from culture-based methods to high throughput sequencing [13, 15, 12, 15–17]. However, data for the human microbiota composition during CDI are still limited and comparison between low and high toxin gene load or between colonizer and overt CDI rarely performed.

The present study aimed to compare the composition of the gut microbiota in healthy controls and *C*. *difficile* toxin positive patients using sequencing of the 16S rRNA gene. We attempted to find key features indicating CDI and to compare the microbiota composition between subgroups based on the toxin gene load and clinical criteria in *C*. *difficile* toxin positive patients.

## Materials and methods

### Clinical samples

This study was approved by the Institutional Review Board of the Konkuk University Medical Center, Seoul, Korea. This study included 99 fecal samples from patients, which were submitted to our center for laboratory tests. These included 79 *tcdB* positive samples by real-time PCR (Xpert *C*. *difficile* system, Cepheid, Sunnyvale, CA, USA) from March 2017 to October 2017 and the other 20 fecal samples were obtained from healthy controls whose samples were submitted for occult blood test of general health examination (controls). This study required neither study-specific nor any other interventions and the data were analyzed anonymously. Therefore, written informed consent from the enrolled patients was waived by the ethics committee.

### Clinical data collection

We collected clinical data through chart review, including demographic data and laboratory data (white cell count, serum creatinine, and albumin concentrations tested within 3 days of fecal sample collection). We obtained the baseline serum creatinine concentrations from tests performed more than 6 months before study entry. The clinical characteristics are described in Table 1.

**Table 1:** Characteristics of study population.

	Control	*tcdB* positive	*P*	low *tcdB*	high *tcdB*	*P*
Number	20	79		49	30	
Female (%)	50 (50%)	41 (51.9%)	1	23 (46.9%)	17 (58.6%)	0.401
Age (years, SD)	62.2 (14.4)	62.5 (19.9)	0.8724	59.9 (19.8)	64.0 (17.5)	0.3538
WBC (10^9^/L, SD)		8,932 (7,886)	-	8,633 (8,804)	9,448 (6,097)	0.6297
50% rise in creatinine (%)		9 (11.4%)	-	5 (10.0%)	4 (13.8%)	1
Albumin (g/dL, SD)		3.3 (0.47)	-	3.3 (0.42)	3.3 (0.55)	0.8095
CDI* (%)		58 (73.4%)	-	40 (80.0%)	18 (62.1%)	0.3904

* (≥ 3 unformed stools in 24 hours)

Abbreviation: CDI, *C*. *difficile* infection

The *tcdB* positive samples were categorized according to *tcdB* gene load (low *tcdB*, n = 49 and high *tcdB*, n = 30) based on the cycle threshold (Ct) values of *tcdB* real-time PCR suggested from previous study [18] and the presence of significant diarrhea; colonizer (< 3 unformed stools in 24 hours, n = 21) and CDI (≥ 3 unformed stools in 24 hours, n = 58)[2, 8].

### Library preparation and sequencing

Bacterial DNA was extracted from 200 mg of stool sample using a QIAamp DNA stool mini kit (Qiagen, Valencia, CA, USA) according to the manufacturer’s protocol. Bacterial 16S rRNA genes were amplified by polymerase chain reaction (PCR) using an Ion 16SMetagenomics Kit (ThermoFisher Scientific, Waltham, MA, USA) according to the manufacturer’s protocol. The kit includes 2 primer tubes and each tube includes 3 primer sets that amplify the hypervariable regions of 16S rRNA (V2, 4, 8 and V3, 6–7, 9, respectively). PCR amplicons were purified using Agencourt AMPure XP beads (Beckman Coulter, Indianapolis, IN, USA). Sequencing libraries were then prepared using an Ion Plus Fragment Library Kit and Ion Xpress Barcode Adapters (ThermoFisher Scientific) according to the manufacturer’s protocol. Prepared libraries were quantified using a High Sensitivity DNA kit on an Agilent 2100 Bioanalyzer (Agilent Technologies, Santa Clara, CA, USA). Template preparation and sequencing were performed using the Ion Chef System and Ion S5 XL system with Ion 530 Chip Kit (ThermoFisher Scientific).

### Data analysis

Sequencing data were analyzed using the Torrent Suite software 5.8.0 (ThermoFisher Scientific) to filter out low quality and polyclonal reads, as well as to trim any adaptor sequences at the 3′ end. After filtering, the sequencing data were demultiplexed and exported as FASTQ files. The FASTQ files were processed using the Quantitative Insights into Microbial Ecology (QIIME) pipeline 1.9.1.[19]. After quality filtering, 10,530,756 sequences were obtained, with a mean of 96,918 sequences per sample (min: 6,183, max: 374,959). Operational taxonomic units (OTUs) were clustered based on 97% sequence similarity with at least 10 identical sequences and assigned against the curated Greengenes v13.8 reference database at the QIIME web site (http://qiime.org/home_static/dataFiles.html). The reference database was modified by excluding both the IDs and sequences of OTUs that are not assigned to a taxonomy level below order. Alpha diversity was assessed by observed OTUs and Chao1 and also included unidentified OTU. Alpha and beta diversity measures were calculated by QIIME [20]. To compare the microbial diversity between samples, qualitative (unweighted UniFrac) and quantitative distances (weighted UniFrac) were calculated. Microbial diversity was visualized using Principal Coordinate Analysis (PCoA) calculated by QIIME. The mean relative abundance in each group was compared at the phylum, family, and genus levels.

### Statistical analysis

The difference between the continuous variables was analyzed using Student’s *t*-test or the Mann–Whitney U test, and that between categorical variables was analyzed using the chi-squared test, Fisher’s exact test, or the McNemar test. The Kruskal–Wallis test and one-way analysis of variance (ANOVA), followed by the Games–Howel's posthoc test, were used to assess the differences between groups. Permutational multivariate analysis of variance (PERMANOVA) analysis between groups was performed using QIIME. Statistical analysis was performed using MedCalc Statistical Software (version 15.8, MedCalc Software, Mariakerke, Belgium) and IBM SPSS Statistics 22.0 (IBM Corporation, Armonk, NY, USA). *P* values less than 0.05 were considered statistically significant.

## Results

### Comparison of alpha diversity of the gut microbiota among the control and *tcdB* positive patients

**Fig 1:**
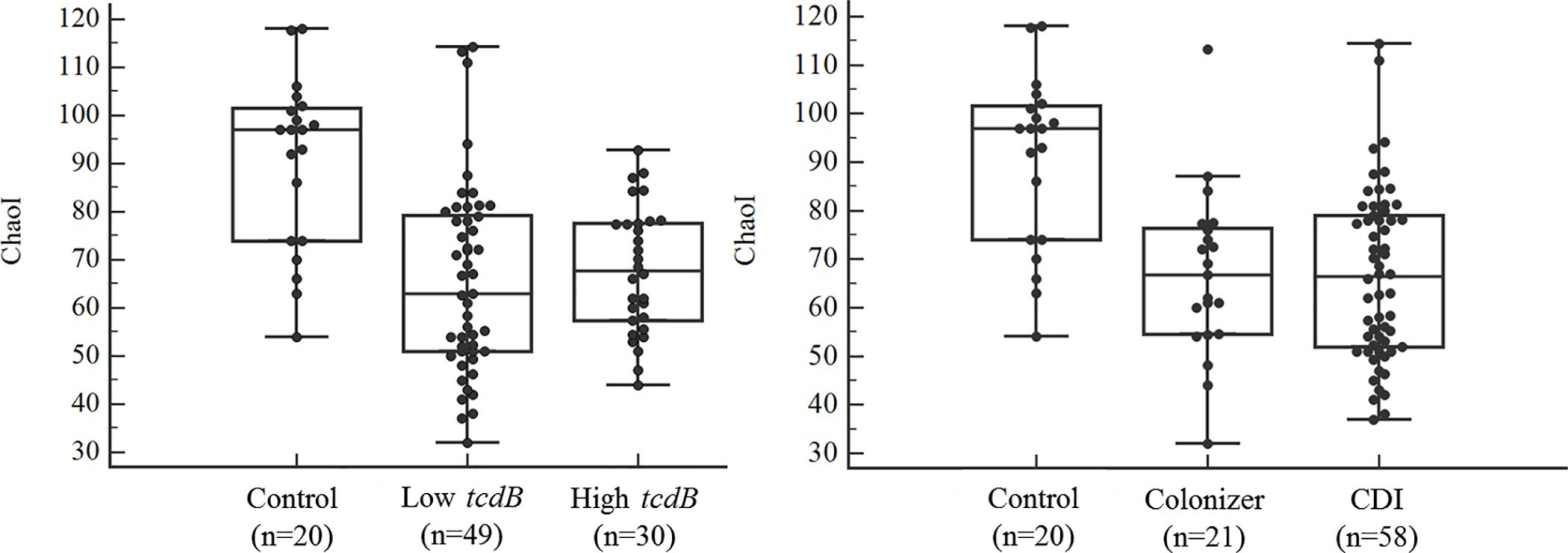
Alpha diversity in control and each category of *tcdB* positive patients. The distribution of ChaoI diversity index was presented between groups (A, control, low and high *tcdB*; B, control, colonizer and CDI, respectively). Black dot line, median value; gray horizontal line, interquartile value.

We evaluated the differences in intra-individual variability (alpha diversity) between the control and each category of *tcdB* positive patients. The distribution of the Chao1 indexes in each group is presented in Fig 1. The mean Chao1 index of the control group was significantly higher than that of the *tcdB* positive patients (*P* < 0.001). The mean Chao1 index between each category of *tcdB* positive patients was not significantly different (*P* = 0.808 and 0.999 between low and high *tcdB* groups and between colonizer and CDI, respectively).

### Comparison of beta diversity of the gut microbiota

**Fig 2:**
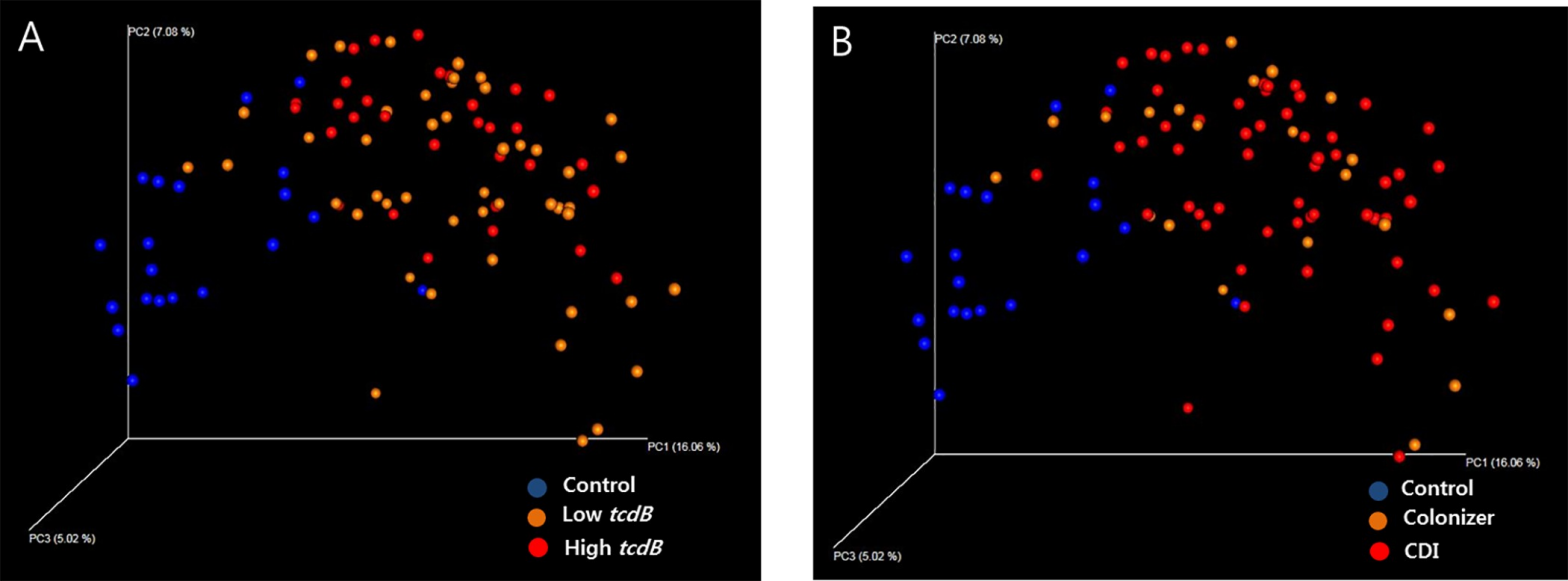
**Evaluation of beta-diversity in control (blue) and *tcdB* positive patients (A, low and high *tcdB*; B, colonizer and CDI, orange and red, respectively).** Principal coordinate analysis (PCoA) was performed using unweighted UniFrac distances of 16S rRNA gene sequences. The each axis represents intersample variation.

Principal Coordinate Analysis (PCoA) using weighted and unweighted UniFrac matrix was performed to evaluate the beta diversity among the samples in each group. In both analysis, the control and *tcdB* positive patients clustered separately (PERMANOVA *P* = 0.001), while the *tcdB* positive patients categorized by *tcdB* gene load (Fig 2A) or presence of significant diarrhea (colonizer vs. CDI)(Fig 2B) could not be separated (only analysis by unweighted UniFrac matrix was shown).

The comparison of mean relative abundance in each group at the phylum level is shown in Table 2. The predominant phyla were *Firmicutes* and *Bacteroidetes* in the control group and *Firmicutes*, *Bacteroidetes*, and *Proteobacteria* in the *tcdB* positive patients. The mean proportion of *Proteobacteria* was significantly higher in the *tcdB* positive patients compared with that in the control group (32.44% *vs*. 21.44%, *P* = 0.008). The mean proportion of *Firmicutes* was significantly lower in the high *tcdB* group compared with that in the low *tcdB* group (27.67% *vs*. 37.90%, *P* = 0.038). The comparisons of mean relative abundance in each group at the family and at genus levels are shown in Tables 3 and 4. In all groups, the *Bacteroidaceae* family was predominant, followed by the *Lachnospiraceae*, *Enterobacteriaceae*, and *Ruminococcaceae*. The *Lachnospiraceae*, *Ruminococcaceae*, and *Prevotellaceae* showed a significantly lower mean proportion in the *tcdB* positive patients compared with that in the control (*P* = 0.003, 0.000, and 0.000, respectively). The *Enterobacteriaceae*, *Porphyromonadaceae*, and *Enterococcaceae* showed a significantly higher mean proportion in the *tcdB* positive patients compared with that in the control (*P* = 0.005, 0.000 and 0.000, respectively)(Table 3). Genera including *Prevotella*, *Phascolarctobacterium*, *Haemophilus*, *Lachnospira*, *Coprococcus*, *Dialister*, *Butyricimonas*, *Catenibacterium*, *Faecalibacterium*, *Paraprevotella*, *Odoribacter*, and *Anaerostipes* were present at a significantly lower proportion in the *tcdB* positive patients compared with that in the control (Table 4). The genera *Parabacteroides*, *Enterococcus*, *Veillonella*, *Klebsiella*, and *Akkermansia* were present at a significantly higher proportion in the *tcdB* positive patients compared with that in the control. The genera *Klebsiella* and *Akkermansia* were present at a significantly different proportion between high *tcdB* group and low *tcdB*, and *Oscillospira* was present at a significantly different proportion between colonizer and CDI.

**Table 2:** Comparison of the mean relative abundance (%) in each group at phylum level.

Phylum	Control (n = 20)	*tcdB* positive (n = 79)	*P*	*tcdB* positive					
				low *tcdB* (n = 49)	high *tcdB* (n = 30)	*P*	colonizer (n = 21)	CDI (n = 58)	*P*
*Firmicutes*	38.73	34.02	0.196	**37.90**	**27.67**	**0.038**	36.30	33.19	0.595
*Bacteroidetes*	36.14	30.26	0.085	27.02	35.56	0.107	28.12	31.03	0.619
*Proteobacteria*	**21.44**	**32.44**	**0.008**	31.71	33.64	0.703	32.48	32.43	0.992
*Actinobacteria*	2.42	1.33	0.254	1.74	0.66	0.115	1.18	1.38	0.830
*Fusobacteria*	0.09	0.73	0.354	0.32	1.39	0.229	0.54	0.79	0.744

Abbreviation: CDI, *C*. *difficile* infection. Phyla with mean relative abundance >1.0 were described in Table 2.

**Table 3:** Comparison of the mean relative abundance (%) in each group at family level.

Family	Control (n = 20)	*tcdB* positive (n = 79)	*P*	*tcdB* positive					
				low *tcdB* (n = 49)	high *tcdB* (n = 30)	*P*	colonizer (n = 21)	CDI (n = 58)	*P*
*Bacteroidaceae*	20.28	22.22	0.545	19.17	27.21	0.093	18.61	23.53	0.353
*Lachnospiraceae*	**16.51**	**9.00**	**0.003**	8.24	10.25	0.399	10.91	8.31	0.319
*Enterobacteriaceae*	**13.75**	**28.09**	**0.005**	27.55	28.98	0.769	27.82	28.19	0.945
*Ruminococcaceae*	**11.39**	**4.22**	**0.000**	3.52	5.36	0.176	6.86	3.27	0.063
*Prevotellaceae*	**8.25**	**1.22**	**0.000**	1.30	1.08	0.404	0.60	1.44	0.528
*Veillonellaceae*	4.40	3.34	0.435	2.43	4.84	0.093	2.77	3.55	0.593
*Alcaligenaceae*	2.91	2.00	0.396	1.39	2.99	0.154	2.60	1.78	0.476
*Pasteurellaceae*	2.23	0.23	0.034	0.17	0.32	0.586	0.01	0.31	0.323
*Bifidobacteriaceae*	2.13	0.53	0.124	0.60	0.43	0.677	0.37	0.59	0.602
*Porphyromonadaceae*	**1.29**	**4.29**	**0.000**	4.35	4.20	0.917	5.10	4.00	0.501
*Streptococcaceae*	1.19	0.68	0.253	0.84	0.40	0.178	0.51	0.74	0.610
*Erysipelotrichaceae*	1.13	1.10	0.938	1.15	1.01	0.755	0.67	1.25	0.233
*Rikenellaceae*	1.10	1.36	0.797	1.02	1.92	0.380	2.23	1.04	0.293
*Lactobacillaceae*	1.07	4.07	0.079	6.50	0.09	0.017	3.31	4.35	0.782
*Enterococcaceae*	**0.56**	**8.70**	**0.000**	**12.87**	**1.89**	**0.001**	8.86	8.65	0.964

Abbreviation: CDI, *C*. *difficile* infection. Familes with mean relative abundance >1.0 or with significant differences were described in Table 3.

**Table 4:** The mean relative abundance (%) of selected genera in each group.

Genus	Control (n = 20)	*tcdB* positive (n = 79)	*P*	*tcdB* positive					
				low *tcdB* (n = 49)	high *tcdB* (n = 30)	*P*	colonizer (n = 21)	CDI (n = 58)	*P*
*Bacteroides*	20.25	22.22	0.539	19.17	27.21	0.093	18.61	23.53	0.353
*Prevotella*	**8.22**	**1.22**	**0.001**	1.29	1.01	0.844	0.60	1.44	0.483
*Sutterella*	2.91	2.00	0.396	1.39	2.99	0.154	2.60	1.78	0.447
*Bifidobacterium*	2.13	0.53	0.124	0.59	0.43	0.678	0.37	0.59	0.603
*Phascolarctobacterium*	**1.97**	**0.55**	**0.033**	0.51	0.63	0.759	0.54	0.56	0.962
*Haemophilus*	**1.96**	**0.21**	**0.040**	0.16	0.29	0.615	0.01	0.29	0.320
*Lachnospira*	**1.59**	**0.37**	**0.004**	0.39	0.34	0.900	0.74	0.24	0.361
*Coprococcus*	**1.29**	**0.48**	**0.008**	0.55	0.37	0.516	0.47	0.48	0.969
*Parabacteroides*	**1.28**	**4.12**	**0.000**	4.29	3.84	0.760	5.04	3.78	0.441
*Oscillospira*	1.22	2.04	0.061	1.39	3.11	0.070	**3.99**	**1.34**	**0.032**
*Streptococcus*	1.17	0.66	0.250	0.83	0.40	0.179	0.51	0.72	0.619
*Dialister*	**0.88**	**0.18**	**0.031**	0.07	0.36	0.092	0.22	0.17	0.743
*Butyricimonas*	**0.64**	**0.18**	**0.009**	0.15	0.21	0.688	0.53	0.05	0.067
*Enterococcus*	**0.56**	**8.70**	**0.000**	**12.8**	**0.89**	**0.001**	8.86	8.65	0.964
*Catenibacterium*	**0.53**	**0.00**	**0.018**	0.00	0.00	-	0.00	0.00	-
*Veillonella*	**0.52**	**2.38**	**0.005**	1.54	3.75	0.115	1.38	2.74	0.331
*Faecalibacterium*	**0.48**	**0.10**	**0.000**	0.10	0.10	0.981	0.09	0.10	0.826
*Paraprevotella*	**0.48**	**0.08**	**0.019**	0.05	0.13	0.361	0.04	0.10	0.567
*Odoribacter*	**0.46**	**0.12**	**0.008**	0.05	0.24	0.216	0.53	0.05	0.419
*Anaerostipes*	**0.38**	**0.01**	**0.019**	0.00	0.02	0.298	0.04	0.00	0.232
*Klebsiella*	**0.05**	**0.75**	**0.000**	0.68	0.89	0.596	0.46	0.85	0.302
*Akkermansia*	**0.01**	**0.14**	**0.025**	**0.01**	**0.35**	**0.022**	0.26	0.09	0.222

Abbreviation: CDI, *C*. *difficile* infection. Only genera with mean relative abundance >1.0 or >0.1 with significant differences were described in Table 4.

The proportion of patients within the control and CDI groups harbouring detectable levels of specific genera are shown in Table 5. *Prevotella*, *Phascolarctobacterium*, *Haemophilus*, *Lachnospira*, *Coprococcus*, *Dialister*, *Butyricimonas*, *Catenibacterium*, *Faecalibacterium*, *Paraprevotella*, *Odoribacter*, and *Anaerostipes* were not detected in a considerable proportion of the CDI group (26.6% to 100.0%); however, proportion of “no detection” were significantly lower in the control group (0.0% to 55.0%).

**Table 5:** The proportion of samples with no detection of several genera in control and CDI.

	Control (n = 20)	CDI (n = 58)	*P*
No detection of			
*Prevotella*	3 (15.0%)	27 (46.6%)	0.0128
*Phascolarctobacterium*	4 (20.0%)	42 (72.4%)	<0.0001
*Haemophilus*	4 (20.0%)	35 (60.4%)	0.0020
*Lachnospira*	1 (5.0%)	45 (77.6%)	<0.0001
*Coprococcus*	0 (0.0%)	21 (26.6%)	-
*Dialister*	6 (30.0%)	44 (75.9%)	0.0002
*Butyricimonas*	3 (15.0%)	50 (86.2%)	<0.0001
*Catenibacterium*	11 (55.0%)	58 (100.0%)	-
*Faecalibacterium*	1 (5.0%)	35 (60.3%)	<0.0001
*Paraprevotella*	9 (45.0%)	51 (87.9%)	0.0001
*Odoribacter*	4 (20.0%)	47 (81.0%)	<0.0001
*Anaerostipes*	4 (20.0%)	57 (98.3%)	<0.0001

## Discussion

The gut microbiota plays a key role in maintaining normal homeostasis by modulating the immune system [21]. An altered intestinal microbiota can result from various influences, including antibiotics, diet, lifestyle, and hygiene. The state of the gut microbiota is also related to certain disease states, especially chronic inflammation or metabolic dysfunction, such as obesity [21]. Disruption of the gut microbiota is a key mechanism of CDI, and a decrease in species abundance and diversity has been consistently observed in previous studies using various methods [8, 11, 15]. However, more data on microbial composition for human CDI is required and comparison between low and high toxin gene load or between colonizer and overt CDI rarely performed. In terms of diagnosis of CDI, qualitative *tcdB* gene positivity by PCR cannot distinguish asymptomatic colonization from symptomatic infection [5]. Recent many studies suggested that toxin gene load (low Ct) as a predictor for free toxin positivity [18, 22–26] although conflicting results also exist on correlation between toxin load and disease outcome [22, 24, 27]. In this study, we categorized *tcdB* positive patients by the *tcdB* gene load and the presence of significant diarrhea (colonizer and CDI), and compared the gut microbiota between them. Moreover, there are very few data on the gut microbiota profile of the Korean population, which might have different dietary habits, such as the consumption of kimchi.

As expected, the alpha diversity index, in this case Chao1, was significantly lower in the *tcdB* positive patients compared with that in the control group. Other studies also showed decreased alpha diversity in CDI or antibiotic exposure group compared with the control [11, 15]. However, the diversity between low and high *tcdB gene* load or between colonizer and CDI showed no significant difference (Fig 1). A study with small study population also showed similar alpha diversity between CDI and asymptomatic colonizers [15]. In this study, colonizer did not meet criteria of significant criteria but they could not be included as healthy population because they were hospitalized patients. Healthy toxin-producing *C*. *difficile* colonizers were not included in our study and could be evaluated in further study. Decreased species abundance and diversity might be features of CDI but could also occur in many hospitalized patients without CDI. The development of overt CDI or more severe disease can be affected by host factors, such as immunity, age, or hospital stay [8, 28]. In this study, alpha diversity analysis also included unidentified OTUs by 97% sequence identity because unidentified OTU should be counted for diversity. OTUs with less than 10 sequences were discarded due to the possibility of error like many previous studies [29, 30]. Chao1 values could be changed whether low-abundance read is included in analysis. We compared Chao1 values between groups and these rules were applied to each group in same conditions. Similarly, PCoA showed evident separation between the control and *tcdB* positive patients, but mixed patterns between low and high *tcdB* gene load or between colonizer and CDI. Data on the comparison between subgroups of *tcdB* positive is lacking and we need to confirm this finding in a further study.

The relative abundance of specific OTUs among the total OTUs showed different trends between the control and *tcdB* positive patients. At the phylum level, compared with the control, *tcdB* positive patients showed a significantly higher mean relative abundance of *Proteobacteria* (*P* = 0.003). Decreased *Bacteroidetes* and increased *Proteobacteria* in CDI have been observed in previous studies [15, 31, 32]. Our results were similar and seem to be recurrent findings in CDI but these features were also observed in colonizer in this study. In this study, the decrease in the abundance of *Bacteroidetes* in the *tcdB* positive patients was not statistically significant (*P* = 0.085), which might be resulted from low statistical power due to the low number of subjects or specific features in our population. Decreased *Bacteroidetes* and increased *Proteobacteria* have been also observed after vancomycin treatment in CDI [17]. Only the *Firmicutes* phylum demonstrated a significantly lower proportion in the high *tcdB* group compared with that in the low *tcdB* group (*P* = 0.038). It could be associated with other bacteria such as *Enterococcaceae*. In contrast to the low *tcdB* group, in the high *tcdB* group, we could assume that *C*. *difficile* replication is high and leads to high toxin production.

At the family level, *Lachnospiraceae*, *Ruminococcaceae*, and *Prevotellaceae* showed significantly lower proportions in *tcdB* positive patients and not significantly different between colonizer and CDI. Decreases in *Lachnospiraceae* and *Ruminococcaceae* have also been reported in other studies [33] and the presence of these families has been shown to correlate with protection against CDI [34]. *Enterobacteriaceae*, *Porphyromonadaceae*, and *Enterococcaceae* were present at a significantly higher proportion in *tcdB* positive patients, and the increased *Enterobacteriaceae* and *Enterococcaceae* agreed with the findings of previous studies [11, 35].

The significant decreases in *Prevotella* and *Faecalibacterium*, and in the genera of the *Lachnospiraceae*, such as *Lachnospira*, *Odoribacter*, *Coprococcus*, and *Anaerostipes* were also important findings in CDI [11, 15]. *Faecalibacterium* and *Bifidobacterium* have health-promoting activities and their low prevalence is associated with many intestinal disorders, such as inflammatory bowel diseases [36, 37]. We observed that many genera of the *Lachnospiraceae*, such as *Lachnospira*, *Odoribacter*, *Coprococcus*, and *Anaerostipes*, a butyrate-producing organism, were present at significantly lower proportions in *tcdB* positive patients. Butyric acid decreases intestinal permeability and improves defense against infection [16, 38]. The changes in the proportions of these genera observed in the colonizer and CDI and did not differ by *tcdB gene* load. This finding suggested that depletion of these health-promoting genera occurred not only in severe disease, but also in mild forms or in various other conditions in hospitalized patients. Several genera, including *Parabacteroides*, *Enterococcus*, *Veillonella*, *Klebsiella*, and *Akkermansia* were present at significantly higher proportions in *tcdB* positive patients. Increased *Parabacteroides*, *Enterococcus*, *Klebsiella*, and *Akkermansia* in CDI have been observed in other studies and reflect a blooming phenomenon resulting from reduced ecological niche competition [11, 15, 39, 40]. However, *Akkermansia* (*A*. *muciniphila*) is associated with a healthier metabolic status in different settings of recent studies [41, 42].

Importantly, many genera were not detected in our analysis platform in most CDI patients; however, “no detection” was rarely observed in the control group (Table 5), especially for *Phascolarctobacterium*, *Lachnospira*, *Butyricimonas*, *Catenibacterium*, *Paraprevotella*, *Odoribacter*, and *Anaerostipes* (*P* < 0.0001) (Table 5). These features could be signature changes of CDI. These changes also occurred in colonizer and could be further studied [34, 43].

This study had several limitations. First, this study could not assess the cause–effect relationship between specific alterations of the microbiota and clinical status. There are also many covariates that could affect the gut microbiota composition [44]. In this study, we simply tried to compare the microbiota composition between subgroups rather than exploring the cause or independent factors responsible for specific alterations of the microbiota. Second, many OTUs did not have a complete taxonomy label at the genus level; for example, many OTUs of the *Lachnospiraceae* family, which is a common feature of this kind of study. Moreover, the results could be different between algorithms or programs used for OTU analysis [44, 45].

In conclusion, there were significant alterations in the alpha and beta diversity in *tcdB* positive patients (both colonizer and overt CDI) compared with those in the control. We identified several changes in the microbiota of CDI that could be further evaluated as predictive markers. Microbiota differences between clinical subgroups of *tcdB* positive patients require further study in larger controlled studies.
